# A ROTEM-guided algorithm aimed to reduce blood product utilization during neonatal and infant cardiac surgery

**DOI:** 10.1051/ject/2023017

**Published:** 2023-06-28

**Authors:** Aymen N. Naguib, Sergio A. Carrillo, Marco Corridore, Amee M. Bigelow, Ashley Walczak, Nguyen K. Tram, Diane Hersey, Mark Galantowicz, Joseph D. Tobias

**Affiliations:** 1 Department of Anesthesiology & Pain Medicine, Nationwide Children’s Hospital and The Ohio State University College of Medicine Columbus Ohio USA; 2 Department of Cardiothoracic Surgery, Nationwide Children’s Hospital Columbus Ohio USA; 3 The Heart Center, Nationwide Children’s Hospital, Department of Pediatrics, The Ohio State University Columbus Ohio USA

**Keywords:** ROTEM^®^, recombinant factor VIIa, prothrombin complex concentrate, bleeding after pediatric cardiac surgery

## Abstract

*Background*: Neonates and infants undergoing cardiac surgery tend to receive high volumes of blood products. The use of rotational thromboelastometry (ROTEM^®^) has been shown to reduce the administration of blood products in adults after cardiac surgery. We sought to develop a targeted administration of blood products based on ROTEM^®^ to reduce blood product utilization during and after neonatal and infant cardiac surgery. *Methods*: We conducted a retrospective review of data from a single center for neonates and infants undergoing congenital cardiac surgery using cardiopulmonary bypass (CPB) from September 2018-April 2019 (control group). Then, using a ROTEM^®^ algorithm, we collected data prospectively between April-November 2021 (ROTEM group). Data collected included age, weight, gender, procedure, STAT score, CPB time, aortic cross-clamp time, volume, and type of blood products administered in the operating room and cardiothoracic intensive care unit (CTICU). In addition, ROTEM^®^ data, coagulation profile in CTICU, chest tube output at 6 and 24 hours, use of factors concentrate, and thromboembolic complications were recorded. *Results*: The final cohort of patients included 28 patients in the control group and 40 patients in the ROTEM group. The cohort included neonates and infants undergoing the following procedures: arterial switch, aortic arch augmentation, Norwood procedure, and comprehensive stage II procedure. There were no differences in the demographics or procedure complexity between the two groups. Patients in the ROTEM^®^ group received fewer platelets (36 ± 12 vs. 49 ± 27 mL/kg, p 0.028) and cryoprecipitate (8 ± 3 vs. 15 ± 10 mL/kg, p 0.001) intraoperatively when compared to the control group. *Conclusion*: The utilization of ROTEM^®^ may have contributed to a significant reduction in some blood product administration during cardiac surgery for infants and neonates. ROTEM^®^ data may play a role in reducing blood product administration in neonatal and infant cardiac surgery.

## Introduction

Our center initially developed blood conservation strategies for the management of patients refusing blood product administration related to religious convictions [1–3]. These strategies have translated into similar techniques to reduce blood administration and blood product utilization in all patients undergoing cardiac procedures [3]. Despite our successes in reducing the incidence and volume of blood product administration in most age and weight groups, neonates and infants undergoing cardiac surgery requiring Cardiopulmonary bypass (CPB), have represented a major challenge in performing bloodless cardiac surgery. A recent retrospective review at our institution demonstrated that bloodless surgery was less likely in children under 6 kg when compared to all other age and weight groups [3].

Many complex patient and procedure-related factors contribute to the increased need for blood products during CPB and surgery for congenital heart disease (CHD) in neonates and infants [4]. The low circulating blood volume in neonates and infants compared to the CPB circuit results in the hemodilution of circulating coagulation factors [5]. In addition, the relatively large surface area of the CPB tubing may result in increased activation of the coagulation cascade and fibrinolysis that impairs platelet function after separation from CPB [6]. Neonates also have reduced coagulation factors and dysfunctional fibrinogen [4, 7, 8]. Additional factors include the inherent need for anticoagulation with unfractionated heparin and varying degrees of hypothermia which both contribute to increased bleeding risk. These variables result in an increased incidence of coagulation disturbances following separation from CPB that may result in the need to transfuse large volumes of blood and blood products with potential deleterious physiologic impact on postoperative outcomes.

In most cases, coagulation disturbances are treated by the administration of multiple volumes of fresh frozen plasma (FFP), platelets, and cryoprecipitate. When these products fail to produce the desired hemostasis, factor concentrates are used. Coagulation Factor VIIa (Recombinant) (Novo Seven Novo Nordisk Inc., Plainsboro, NJ, USA) (rFVIIa) remains the most widely used factor concentrate both in adult and pediatric cardiac surgery [8–10]). Other factor concentrates, such as prothrombin complex concentrates (PCCs) used in adult cardiac surgery [11], are utilized infrequently in pediatric cardiac surgery [7, 10] as the safety profile of these agents is not established in children. Hence, these factors must be used judiciously and considered as a last step for hemostatic therapy [7, 12].

The use of rotational thromboelastometry (ROTEM^®^, TEM International, Munich, Germany) has proven effective in some adult studies, demonstrating a reduction in blood product transfusion after cardiac surgery utilizing CPB [13–16]. ROTEM is a method to measure hemostasis quality via the viscoelastic properties of the blood clot. ROTEM also provides a rapid assessment of clot development from secondary hemostasis to clot lysis, including clot formation, clot firmness, and clot fibrinolysis. ROTEM provides information beyond the normal testing of the coagulation cascade such as prothrombin time (PT), partial thromboplastin time (PTT), and international normalized ratio (INR). There are different assays included in the ROTEM measurements which target specific components of the coagulation cascade. For example, INTEM is an assay that measures intrinsic pathway activation via ellagic acid. This assay is sensitive to the presence of heparin in the sample. When heparin is present in the sample, the HEPTEM assay is used, in which heparinase is added to the sample, resulting in the removal of up to 7 units/mL of heparin. The EXTEM assay measures the extrinsic pathway of the coagulation cascade. The FIBTEM assay utilizes cytochalasin D to inhibit platelets contribution to the clot formation and the APTEM assay uses antifibrinolytic to inhibit hyperfibrinolysis [17]. Table 1 shows a basic explanation of different ROTEM parameters. In addition, while all the ROTEM^®^ data are extrapolated from adult values, Table 2 shows Nationwide Children’s reference range for the different assays included in ROTEM^®^.

**Table 1:** Basic ROTEM parameters.

Parameter	Description
CT (Clotting time)	Onset of clot formation in seconds
CFT (Clot propagation rate)	Describes generation of thrombin and clot formation in seconds
α angle	Degree of clotting curve in degrees
A10	Amplitude 10 min from clotting in mm
A20	Amplitude 20 min from clotting in mm
MCF (Maximum clot firmness)	Describes the firmness and stability of the clot
ML (Maximum lysis)	Percentage of lysis in (%)

**Table 2:**
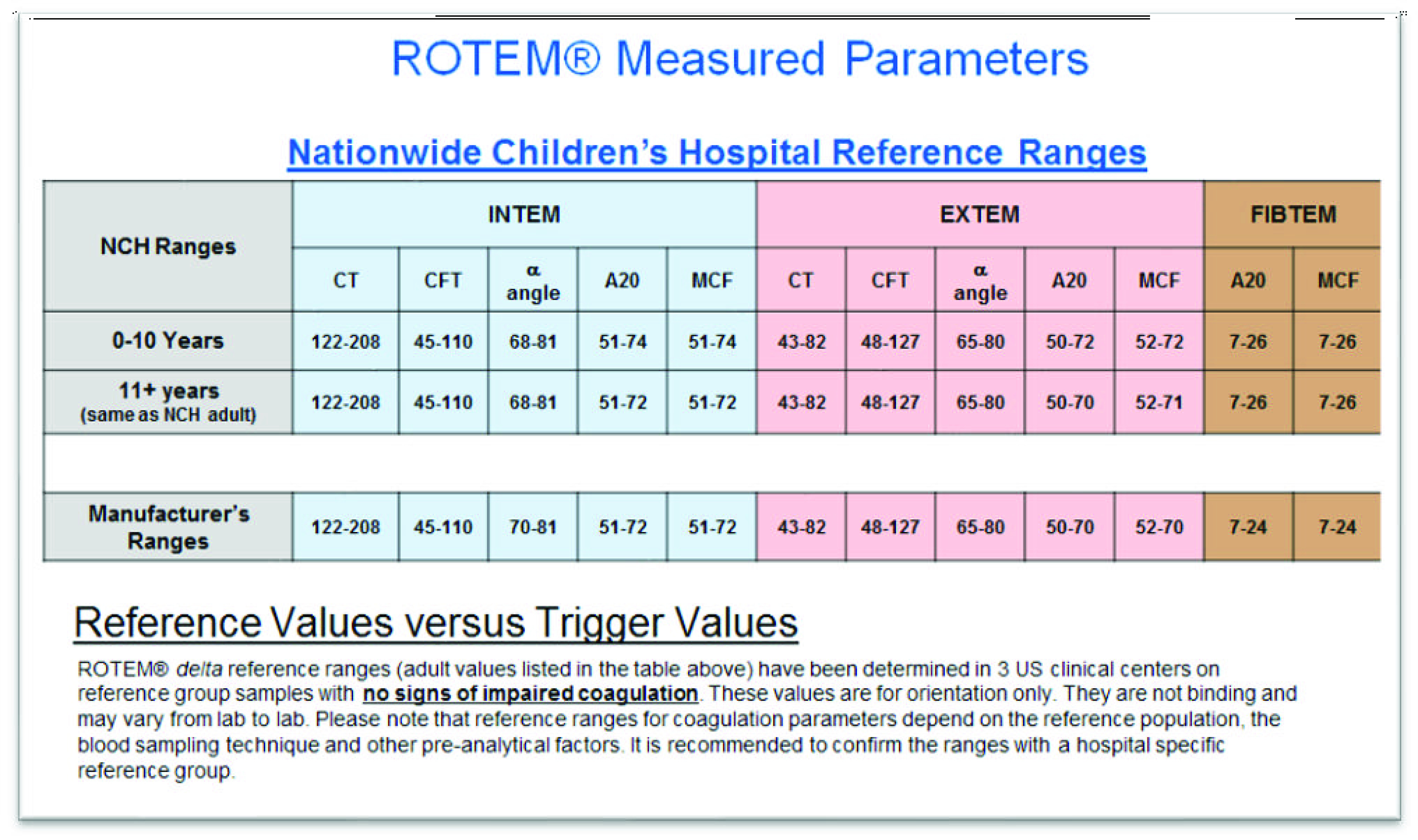
Reference values for ROTEM at NCH.

Although its use in the pediatric population is not widespread, Romlin et al. and Faraoni et al. have attempted to formulate an algorithm using ROTEM^®^ to guide blood product administration [18, 19]. Romlin developed a CPB ROTEM-guided protocol that was associated with a reduction of packed red blood cells (PRBCs) and FFP, and the increased use of platelets and fibrinogen concentrates in the operating room. Faraoni, in univariate and multivariate analysis of the ROTEM^®^ data, revealed EXTEM CT, A10, and FIBTEM A10 to be the three relevant parameters to guide hemostasis following CPB. The authors demonstrated the need to combine this protocol with the probability of bleeding risk to avoid unwarranted transfusions.

We retrospectively identified patients with the highest incidence and volume of blood transfusion requirements, which included neonates and infants undergoing arterial switch procedures, aortic arch augmentation, truncus arteriosus repair, and comprehensive stage II procedures. Our specific aim was a reduction of blood product utilization and use of factors concentrate by targeted administration of blood products based on ROTEM^®^ during these high-risk procedures. This would ultimately help reduce the risk of thromboembolic events.

## Materials and methods

As this was a quality improvement initiative, the need for Institutional Review Board (IRB) approval was not necessary and therefore no number was assigned. Our specific aim was to reduce blood product administration in neonates and infants undergoing surgery for CHD utilizing CPB using ROTEM^®^ as a guide for blood product administration. We retrospectively reviewed neonates and infants undergoing cardiac surgery using CPB between September 2018 and April 2019 to determine patients at the highest risk for bleeding and blood transfusion requirements. These patients were the procedure and age-matched historical controls (control group). For the control group, blood product administration was based on routine clinical practice at perioperative team discretion and was not guided by ROTEM^®^. Intraoperatively and after separation from CPB, we routinely ordered 20–40 mL/kg of platelets, 1–3 units of cryoprecipitate, and 5–10 mL/kg of FFP. These volumes did not account for fresh frozen plasma use during CPB. If bleeding continued after administration of the mentioned products, a second round of these products was transfused in addition to a dose of rFVIIa of 90 mcg/kg. If necessary, a third round of products was administered with a second dose of rFVIIa alone or in combination with factor eight inhibitor bypassing activity (FEIBA, Baxter Healthcare Corp.) at a dose of 10–20 unit/kg or Prothrombin Complex Concentrate (Human), Kcentra^TM^ at a dose of 20 unit/kg.

**Figure 1:**
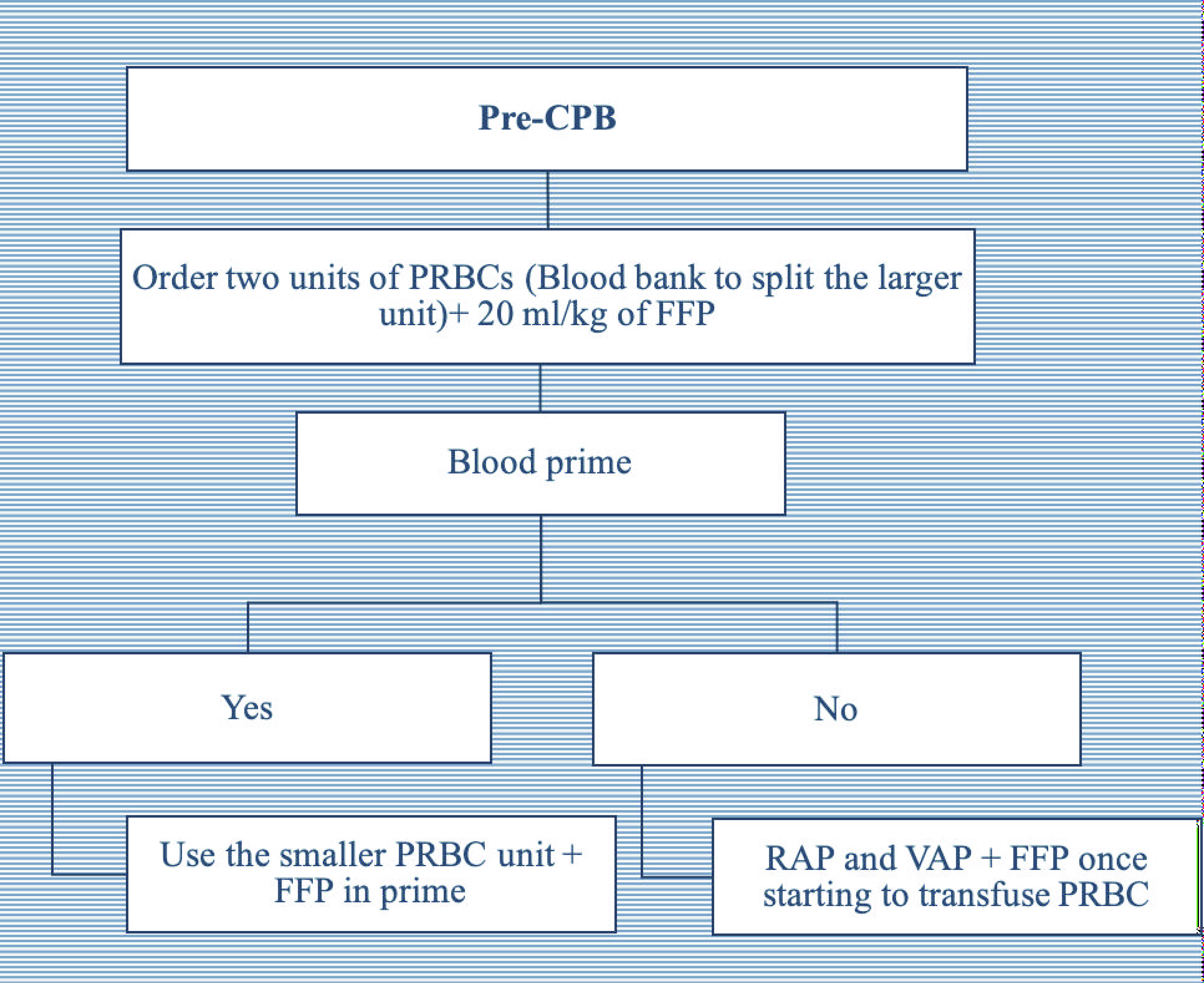
Pre-cardiopulmonary bypass (CPB) algorithm. FFP – fresh frozen plasma; PRBC – packed red blood cells; RAP = retrograde autologous priming; VAP – venous antegrade priming.

**Figure 2:**
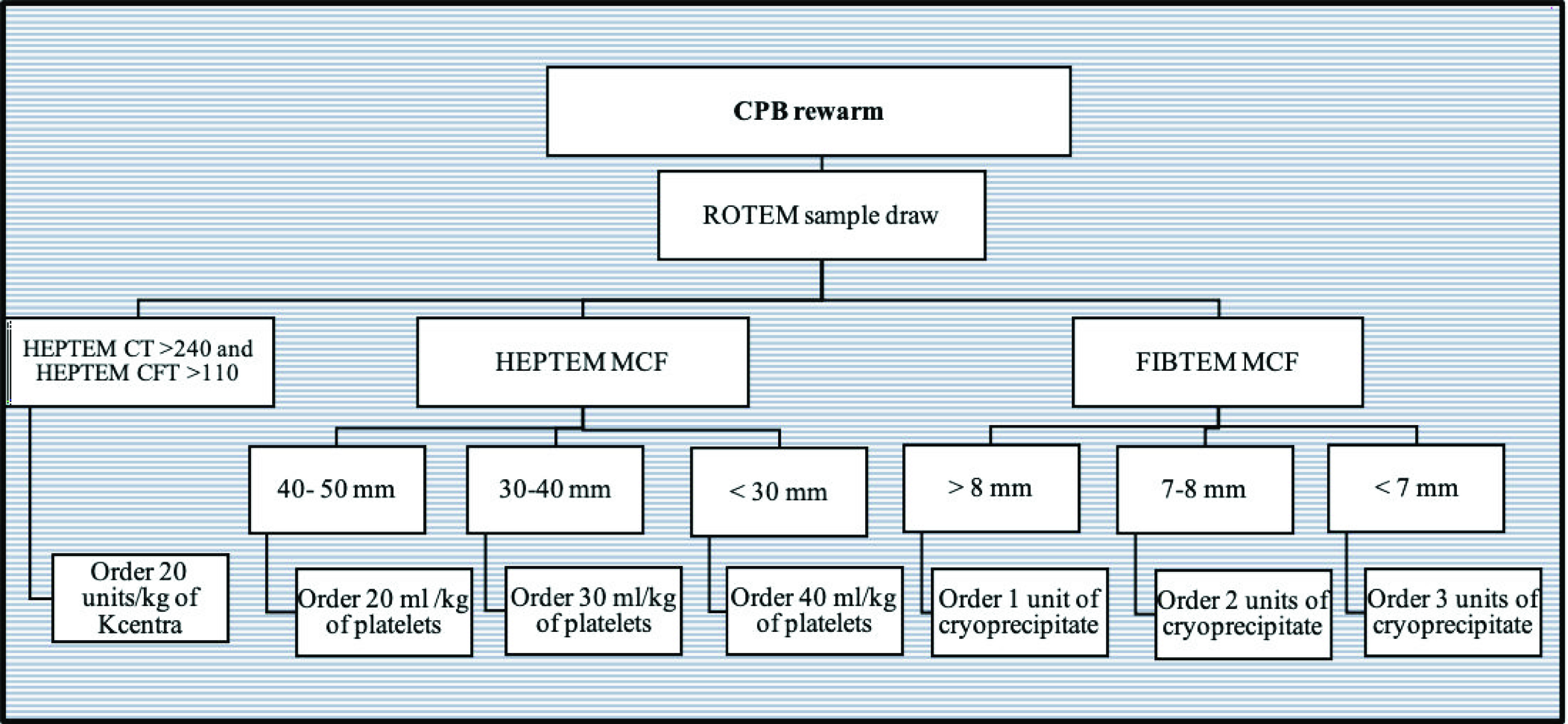
Cardiopulmonary bypass intraoperative algorithm.

**Figure 3:**
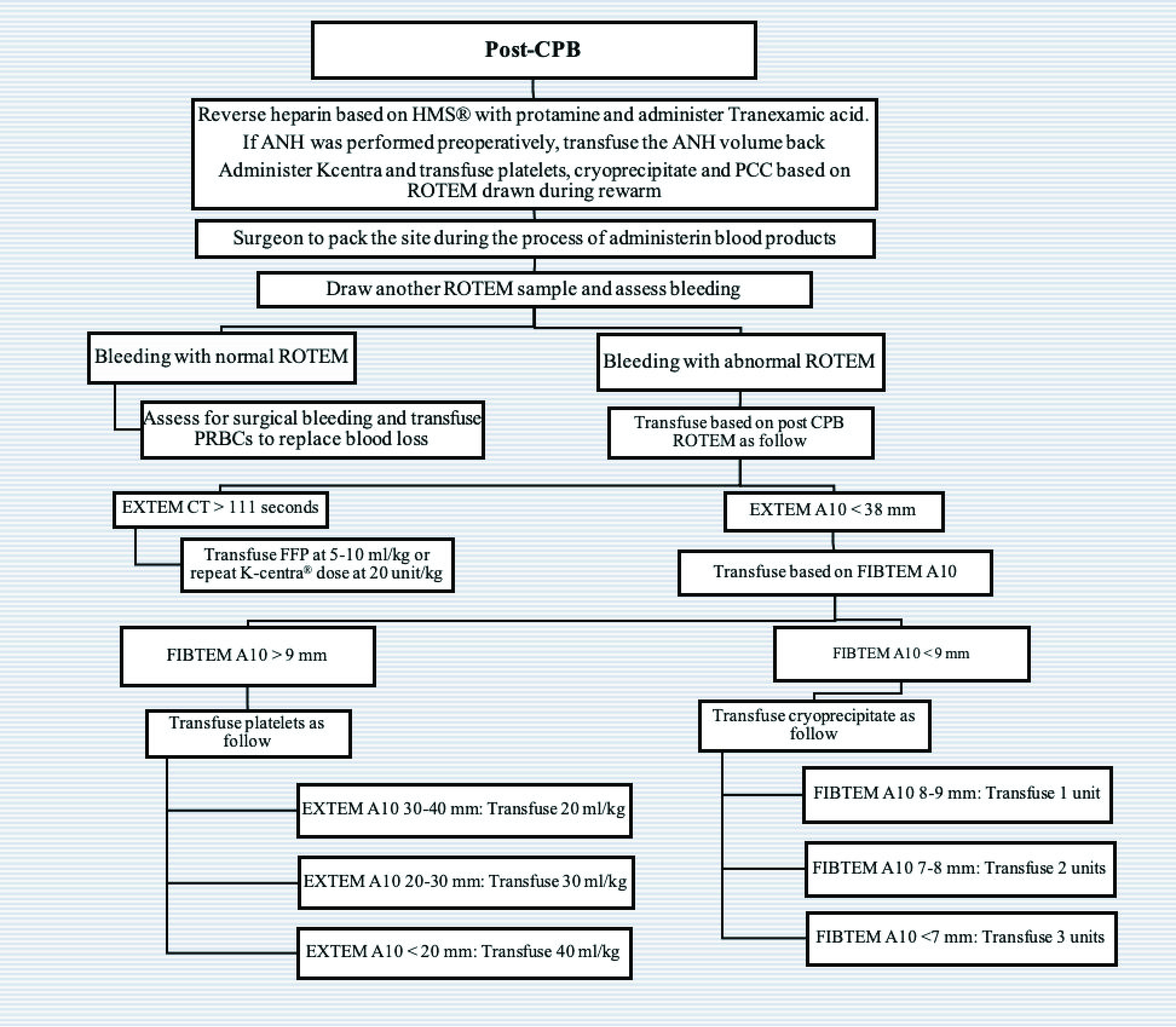
Post-cardiopulmonary bypass algorithm.

Following this retrospective review, we identified a number of high-risk procedures that were associated with a high volume of blood and blood product administration. Over the next period of time, we tested the feasibility of establishing a new protocol using ROTEM^®^ to guide blood product administration during these high-risk procedures. We investigated the specific timing of sending the ROTEM^®^ samples during CPB to the blood bank, the turnaround time between obtaining different results, and the arrival of blood products and factors from the blood bank and pharmacy. Finally, we held multiple meetings to ensure support from the entire team including cardiac surgeons, nurses, perfusionists, the blood bank, and the CTICU team. These efforts finally resulted in a series of perioperative blood and products administration algorithms (Figures 1, 2 and 3) using ROTEM^®^. We then prospectively applied these algorithms to guide blood product administration during pre-determined high-risk neonatal and infant cardiac surgery cases over the period of April 2021–November 2021 (ROTEM group).

Demographic, surgical, and post-operative characteristics were collected. Collected data included age, weight, gender, primary procedure, The Society of Thoracic Surgeons-European Association for Cardio-Thoracic Surgery (STAT) score, CPB time, aortic cross-clamp time, volume and type of blood products administered in the operating room and Cardiothoracic Intensive Care Unit (CTICU). In addition, ROTEM^®^ data, coagulation profile upon arrival to the CTICU, chest tube output at 6 and 24 h, and the use of factor concentrates were collected. Thromboembolic complications following product administration were also recorded.

### Perioperative considerations

#### Procedural planning

The day prior to surgery, the perioperative team, including critical care, cardiac surgery, perfusion, cardiology, and cardiac anesthesia held meetings to discuss and highlight potential challenges. The team formulated a plan for preoperative optimization, intraoperative management, surgical repair, and immediate postoperative course including the need for mechanical ventilation and inotropic support.

A preoperative multidisciplinary huddle was conducted before the arrival of the patient to highlight specific intraoperative details. During this time, requirements for invasive monitoring, cannulation sites, management of CPB, post-bypass inotropic support, chest closure, ventilatory support, and blood conservation management planning were discussed.

#### Intraoperative anesthetic management

In addition to the standard American Society of Anesthesiology monitors, bilateral near-infrared spectroscopy (NIRS) monitoring was used. Induction technique and medications were determined at the discretion of the anesthesiologist and the trachea was intubated. Arterial and central venous lines, as well as peripheral venous lines, were placed under sterile conditions. The anesthetic was maintained using isoflurane in a mixture of air and oxygen, lesion permitting, with 10–15 μg/kg of fentanyl prior to incision. Following induction, a dexmedetomidine infusion was started at 0.5–1 μg/kg/h. Dexmedetomidine was maintained until separation from CPB. All patients received tranexamic acid at a dose of 20 mg/kg in three doses; prior to incision, during CPB, and after separation from CPB and reversal of heparin using protamine.

#### Acute normovolemic hemodilution (ANH)

Depending on the nature of the lesion, the overall physiologic status of the patient, and the estimated hematocrit during CPB, the decision to draw off blood pre-incision for autologous transfusion post-bypass was made. Our goal for patients weighing greater than 5 kg was to draw 10–20% of the total estimated patient blood volume. For patients weighing less than 5 kg, we attempted to withdraw 10–20 mL/kg. The whole blood (ANH) was removed using the patient’s arterial line into a syringe with 7 mL of Anticoagulant Citrate Dextrose Solution USP Formula A (ACD-A) (Fenwal, Inc., Lake Zurich, IL, USA) per every 50 mL of blood. The ANH blood was stored at room temperature in the Operating Room [3, 20]. Bolus doses of phenylephrine (0.5–1 μg/kg) or epinephrine (0.5–1 μg/kg) were administered as needed to achieve physiologic stability [3, 20, 21].

#### Cardiopulmonary bypass

Depending on the weight and clinical status of the patient, the CPB circuit was either blood or crystalloid primed. In the case of a crystalloid prime, and after arterial and venous cannula placement, we attempted retrograde arterial priming (RAP) and venous autologous priming (VAP) to displace all crystalloid volume from the perfusion circuit. During RAP and VAP, bolus doses of phenylephrine (0.5–1 μg/kg) were administered to maintain hemodynamic stability [3, 20, 21].

#### Perfusion technique

CPB circuits used for all patients in the study consisted of 1/8″ × 3/16″ Xcoating™ (Terumo Cardiovascular, Ann Arbor, MI, USA) AV loop with a raceway size of ¼′ × 3/32′ for a total circuit prime volume of 300 mL. Capiox^®^ FX05 Oxygenator (Terumo Cardiovascular, Ann Arbor, MI, USA) and CSC14 Cardioplegia Heat Exchanger (Liva Nova PLC, London, UK) were added to the CPB machine. Moderate hypothermia (24–25 °C) was used in all cases. A Continuous Autotransfusion device (C.A.T.S. ^®^ *Plus*) (Fresenius Kabi AG, Bad Homburg, Germany) was utilized in all cases.

#### Anticoagulation during CPB

Heparin (Hospira, Forest Lake, IL, USA) was used as the anticoagulant for all cases. The HMS^®^ Plus (Medtronic, Minneapolis, MN, USA) was utilized to measure the heparin dose response (HDR) and the activated clotting time (ACT), both of which guided heparin administration throughout CPB.

#### Post-CPB anesthetic management

Inotropic support was prescribed as needed, and heparin was reversed with protamine based on HMS data. Once the patients’ hemodynamics were satisfactory and hemostasis was achieved, the surgeon proceeded with chest closure. Immediate or fast-track (<6 h) tracheal extubation was considered in all patients as per routine clinical practice [21, 22]). If there were any concerns for further mediastinal bleeding or hemodynamic instability, extubation was not attempted and sedation was maintained for transport to the CTICU.

### Intraoperative blood transfusion plan

#### Pre-CPB blood management

(Figure 1) Two units of PRBCs were ordered. To reduce blood waste, we requested the blood bank to split the larger of the two PRBCs units into two aliquots. The smaller unit was used first for blood prime or transfusion during CPB. In addition, 20 mL/kg of FFP was added to the blood prime or once PRBCs were transfused during CPB.

#### CPB ROTEM

The ROTEM^®^ sample was drawn once rewarming was started. Based on the results obtained from the sample, the anesthesiology team ordered blood products and PCCs for use after separation from CPB (Figure 2).

#### Post-CPB

After reversal of heparin and administration of tranexamic acid, if ANH was performed, the ANH volume was transfused back to the patient. This was followed by the administration of platelets, cryoprecipitate, and PCCs based on CPB ROTEM^®^ data. The surgeon packed the operative site while administering the blood products to assist in hemostasis. After blood products administration, another ROTEM^®^ sample was drawn and further blood product was administered based on the status of bleeding and the ROTEM^®^ data. If surgical bleeding continued and ROTEM^®^ was not corrected, further transfusion requirements were based on the algorithm in Figure 3.

### CTICU care

The standard institutional practice was tracheal extubation in the operating room [22–24], or early tracheal extubation, defined as within 24 h of admission to the CTICU. Upon the patient’s arrival at the CTICU, a postoperative ROTEM^®^ sample was obtained simultaneously with the first postoperative laboratory blood draws to guide further blood transfusions. Postoperative bleeding was defined as total blood loss of >5 mL/kg/h, or between 3–4 mL/kg/h associated with hemodynamic instability. If mediastinal bleeding continued and ROTEM^®^ results were abnormal, blood product transfusion followed the algorithm in Figure 3. Additionally, our standard of care was to transfuse 15–20 mL/kg PRBCs to correct for hemoglobin <7 mg/dL. In the event of ongoing significant bleeding with a normal ROTEM^®^, the operating surgeon assessed the need for re-exploration.

### Statistical analysis

Continuous data are presented as means and standard deviations, while categorical data are presented as frequencies and percentages. Two-sample unequal-variance t-test (Welch’s t-test) for independent samples was used to compare the two groups. A *p*-value < 0.05 was considered statistically significant.

## Results

The study cohort included 24 patients in the control group and 40 in the ROTEM^®^ group. During the early phase of the study, four patients did not follow the ROTEM^®^ protocol due to surgeon’s preference and they were included in the control group, bringing the total number of patients in the control group to twenty-eight. There were no differences in the demographics, the type of procedure performed, STAT scores, CPB time, and aortic cross-clamp time between the two groups (Table 3). Patients in the ROTEM^®^ group received fewer platelets intraoperatively (36 ± 12 vs. 49 ± 27 mL/kg, *p* = 0.028). The ROTEM group received less cryoprecipitate intraoperatively (8 ± 3 vs. 15 ± 10 mL/kg, *p* = 0.001). Patients in the ROTEM^®^ group received more FFP during CPB (33 ± 19 vs. 24 ± 9 mL/kg, *p* = 0.015), with patients in the control group receiving more FFP after CPB (9 ± 15 vs. 0 mL/kg). Ultimately, there was no difference between the two groups in terms of total PRBCs and FFP administered intraoperatively (Table 4).

**Table 3:** Demographics and clinical data.

Variable assessed	Control group	ROTEM^®^ group	*P*-value
Number	28	40	
Age (days)	52 ± 68	44 ± 90	0.6
Weight (kilograms)	4 ± 2	4 ± 2	0.5
Gender (male/female)	18/10	24/16	0.7
STAT score	3 ± 1	3 ± 1	0.8
Procedure			
Aortic arch augmentation	8	14	
Arterial switch operation	5	11	
Norwood	1	1	
Comprehensive stage 2	7	4	
Truncus arteriosus communis	4	4	
TAPVC	1	4	
Other	2	2	
Preoperative hematocrit (%)	40 ± 7	40 ± 6	0.88
Preoperative platelets count (1,000/uL^3^)	267 ± 99	306 ± 95	0.12
CPB time (minutes)	177 ± 55	163 ± 68	0.3
Aortic cross-clamp time (minutes)	64 ± 58	65 ± 54	0.9

Data presented as the mean ± SD or number. STAT: Society of Thoracic Surgeons-European Association for Cardio-Thoracic Surgery; TAPVC: Total anomalous pulmonary venous connection; CPB: cardiopulmonary bypass; ROTEM^®^: rotational thromboelastometry.

**Table 4:** Intraoperative blood products administration.

Variable assessed	Control group	ROTEM group	*P*-value
Intraoperative platelets (mL/kg)	49 ± 27	36 ± 12	0.028
Intraoperative cryoprecipitate (mL/kg)	15 ± 10	8 ± 3	0.001
Intraoperative CPB plasma (mL/kg)	24 ± 9	33 ± 19	0.015
Intraoperative total plasma (mL/kg)	33 ± 18	33 ± 19	0.9
Intraoperative PRBCs (mL/kg) (SD)	99 ± 45	85 ± 39	0.18

Data presented as the mean ± SD. CPB: cardiopulmonary bypass; SD: standard deviation; ROTEM^®^: rotational thromboelastometry; PRBCs: packed red blood cells.

There were no differences between the two groups regarding the preoperative hematocrit (40 ± 6% vs. 40 ± 7%, *p* = 0.8), platelet count (306 ± 95 10^*3^/uL vs. 267 ± 99 10^*3^/uL) (Table 3) and preoperative ROTEM^®^ profiles (Table 5). ROTEM^®^ samples during rewarming on CPB and after blood products administration showed an improved ROTEM^®^ profile in the ROTEM^®^ group (Table 5).

**Table 5:** Rotational thromboelastometry data.

	Control group	ROTEM^®^ group	*P*-value
Baseline EXTEM CT (seconds)	61 ± 11	56 ± 9	0.07
Baseline EXTEM A10 (mm)	54 ± 9	56 ± 8	0.5
Baseline FIBTEM A10 (mm)	15 ± 5	16 ± 6	0.67
CPB HEPTEM CT (seconds)	373 ± 299	286 ± 41	0.25
CPB HEPTEM CFT (seconds)	352 ± 196	233 ± 123	0.03
CPB HEPTEM MCF (mm)	39 ± 7	44 ± 8	0.019
CPB FIBTEM MCF (mm)	6 ± 2	9 ± 3	0.013
Post products EXTEM CT (seconds)	96 ± 26	71 ± 14	0.00007
Post products EXTEM A10 (mm)	41 ± 13	50 ± 8	0.002
Post products FIBTEM A10 (mm)	11 ± 6	14 ± 5	0.008

CPB: cardiopulmonary bypass; ROTEM^®^: rotational thromboelastometry.

**Table 6:** Cardiothoracic Intensive Care Unit transfusion data.

Variable assessed	Control group	ROTEM^®^ group	*P*-value
Packed RBCs (mL/kg)	(10) 53 ± 50	(3) 37 ± 31	0.01
Platelets (mL/kg)	(10) 25 ± 20	(3) 27 ± 21	0.02
Cryoprecipitate (mL/kg)	(7) 15 ± 13	(1) ± 4	0.01
Fresh frozen plasma (mL/kg)	(11) 24 ± 19	(2) 16 ± 8	0.002
Chest tube output 6 h (mL/kg)	29 ± 35	10 ± 8	0.009
Chest tube output 24 h (mL/kg)	41 ± 34	23 ± 15	0.014
First hematocrit (%)	38 ± 9	43 ± 6	0.014
First platelet count (1000/uL^3^)	208 ± 88	220 ± 103	0.6

The data listed as number (*N*) and mean ±SD. CTICU: Cardiothoracic Intensive Care Unit; PRBCs: packed red blood cells; SD: standard deviation.

Patients in the control group received rFVIIa more frequently than the ROTEM^®^ group (12/28, 42.9% vs. 11/40, 27.5%) and at higher doses (143 ± 46 vs. 65 ± 30 mcg/kg, *p* < 0.0001). Patients in the control group tended to receive two or more doses of rFVIIa (7/28, 25% vs. 0/40, 0%) compared to the ROTEM^®^ group. In addition, patients in the control group had a higher incidence of multiple PCC administration. The ROTEM^®^ group received more Kcentra^TM^ than the control group (37/40, 92.5% vs. 3/28, 10.7%, respectively) (Table 7). Patients in the control group tended to receive more PRBCs, platelets, and cryoprecipitate in the CTICU and had increased chest tube output for the first 6 and 24 h postoperatively (Table 6). Patients in the ROTEM^®^ group had a higher hematocrit upon arrival to the CTICU compared to the control group (43 ± 6 vs. 38 ± 9%, respectively, *p* < 0.014) (Table 6). There was no difference between the two groups regarding the coagulation profile using prothrombin time (PT), partial thromboplastin time (PTT), or international normalized ratio (INR) upon arrival at the CTICU.

**Table 7:** Prothrombin complex concentrates (PCC) administration.

	Control group	ROTEM group	*P*
rFVIIa (*N*) (ug/kg)	(12) 143 ± 46	(10) 65 ± 30	0.0001
FEIBA (*N*) (ut/kg)	(7) 37 ± 33	0	0.02
Kcentra^TM^ (*N*) (ut/kg)	(3) 37 ± 21	(37) 22 ± 12	<0.00001

rFVIIa: activated recombinant factor VII; FEIBA: factor eight inhibitor bypass activity; Kcentra^TM^: prothrombin complex concentrate (human).

Ten patients developed thrombosis after surgery, with five patients in each group. All incidents were detected between 0 and 60 days post-procedure. The use of a combination of multiple-factor concentrates was the common denominator with these thrombotic events in all ten patients. A peripherally inserted central catheter (PICC) or cardiac catheterization access prior to the procedure was correlated with clot formation in 3/5 patients for each group (Table 8).

**Table 8:** Detailed description of thromboembolic events.

Age (days)	Procedure	Site of thrombus	Time of thrombus (days after procedure)	Line associated	Factors used and dose
Control group					
4	Aortic arch repair	Left subclavian vein	0	No	Kcentra^TM^ (20 ut/kg) + FEIBA (50 ut/kg) + rFVIIa (90 ug/kg)
6	Arterial switch	IVC	8	UVC	Kcentra^TM^ (30 ut/kg) + rFVIIa (90 ug/kg)
165	Comprehensive stage II	Venous brain infarct, stroke, seizures	2	No	FEIBA (100 ut/kg) + rFVIIa (180 ug/kg)
98	Truncus arteriosus repair	Right common femoral vein	60	S/P previous cardiac catheterization access	FEIBA (50 ut/kg) + rFVIIa (90 ug/kg)
10	MAPCAs unifocalization	Left common femoral vein	10	PICC line	FEIBA (50 ut/kg) + rFVIIa (90 ug/kg)
ROTEM group					
5	Arterial switch	IVC (near RA junction)	8	PICC line	Kcentra^TM^ + rFVIIa (45 ug/kg)
8	Arterial switch	Right superior femoral artery and vein	20	Cardiac Cath access	Kcentra^TM^ + rFVIIa (45 ug/kg)
141	Comprehensive stage II	LPA thrombus	8	No	Kcentra^TM^ + rFVIIa (45 ug/kg)
150	Comprehensive stage II	LPA thrombus	11	No	Kcentra^TM^ + rFVIIa (45 ug/kg)
1	TAPVC	Right common femoral and external iliac vein	18	Cardiac Cath access	Kcentra^TM^ + rFVIIa (45 ug/kg)

MAPCA’s: Major aortopulmonary collateral arteries; TAPVC: total anomalous pulmonary venous connection; rFVIIa: activated recombinant factor VII; FEIBA: factor eight inhibitor bypass activity; Kcentra^TM^: prothrombin complex concentrate (human); LPA: left pulmonary artery; IVC: inferior vena cava.

## Discussion

The main finding of this study was that the utilization of a novel algorithm showed that a targeted transfusion approach may have contributed to a reduction in overall blood product administration, a better coagulation profile, higher hematocrit, and overall, less bleeding post-operatively in neonates and infants undergoing high-risk cardiac procedures using CPB. The algorithm developed in this study represented an attempt to address post-operative mediastinal bleeding and manage coagulopathy following neonatal and infant cardiac surgery requiring CPB. The use of ROTEM^®^ as a guide to blood products administration offered a possible valuable alternative to the empiric use of blood products and PCC. In some instances, utilization of this protocol was associated with decreased use of certain products like platelets, cryoprecipitate, and rFVIIa. However, this was not the case for FFP, as there was no difference in the total volume of FFP administered between the two groups. This similarity was expected since the volume of FFP administered was not based on ROTEM^®^ guidance, but rather a predetermined dose of FFP transfused during CPB. Intriguing, although speculative, is that administering more FFP during CPB in the ROTEM^®^ group may have resulted in a better coagulation profile after separation from CPB, as evidenced by the significantly better CPB HEPTEM CFT, MCF, and FIBTEM MCF in the ROTEM^®^ group (Table 5). This may have resulted in achieving a better state of hemostasis and ultimately a much better ROTEM^®^ profile after the first round of PCC, platelets, and cryoprecipitate in the ROTEM^®^ group. These findings are in contradiction to other studies suggesting a lack of benefit of prophylactic use of FFP during blood prime [25, 26]. Another point of interest is that, while there was no difference in the total volume of PRBCs transfused in both groups, the hematocrit of patients in the ROTEM^®^ group upon arrival to the CTICU was significantly higher. The higher hematocrit in the ROTEM^®^ group suggests a more aggressive PRBCs transfusion practice. We speculate that, as more confidence is gained using ROTEM^®^ as a guide for blood product administration, we could see a reduction in unnecessary transfusions, fewer thromboembolic events, and an improved cost benefit.

Intraoperatively, targeted administration of blood products based on ROTEM^®^ data was associated with less use of platelets and cryoprecipitate while achieving a better ROTEM^®^ coagulation profile. In addition, postoperatively, the ROTEM^®^ group had a lower chest tube output at 6 and 24 h. Continued use of ROTEM^®^ to guide blood product administration in the CTICU was associated with a lower incidence and volume of blood product administration.

While factors concentrates were used in both groups, the administration of multiple factors was significantly less in the ROTEM^®^ group. It is worth mentioning that, in both groups, the incidence of thrombosis postoperatively was mainly associated with multiple factors administration. This is in line with the CCAS and the Network for Advancement of Patient Blood Management (NATA) task force recommendation against the use of these factors in pediatric cardiac surgery unless it is part of a clinical trial or in the presence of extreme bleeding not responding to standard products [27].

As we are gaining more confidence with this ROTEM^®^ guided protocol, our ultimate goal is to prospectively use it to achieve a much tighter volume of blood product administration. Ultimately, a multicenter randomized study comparing the use of this protocol to the common practice of ordering blood products based on the clinician’s discretion would be the ideal objective. Unfortunately, this goal might be challenging to implement because of the differences in perioperative surgical approaches between various institutions.

## Study limitations

There are some limitations to the study, including the retrospective nature of the historical controls, lack of randomization, and a small number of patients in each group which limit the generalizability of our results, despite homogenization of surgical, anesthetic, and perfusion techniques during the period of the study. We also did not evaluate the cost-benefit for each group.

## Conclusions

In this study, the use of a ROTEM^®^ guided transfusion algorithm may have contributed to a reduction of blood product administration following cardiac surgery for neonates and infants, with better hemostasis postoperatively. The results from this study have helped our team to gain confidence in the management of mediastinal bleeding and severe coagulopathy after cardiac procedures requiring CPB in neonates and infants.

## Data Availability

The research data associated with this article are included within the article.
